# COVID-19, Policy, and Nursing Homes: A Health and Aging Policy Fellows Symposium

**DOI:** 10.1093/geroni/igab046.222

**Published:** 2021-12-17

**Authors:** Nancy Kusmaul, Toni Miles, Lori Frank

## Abstract

COVID-19 significantly impacted older adults, particularly those in long term care. This symposium focuses on policy, and how policies drove many of the outcomes older adults in care experienced during 2020. We begin with a case study of one nursing home describing their operations, how those were impacted by policies at the local, state and federal levels, and operational factors that proved uncontrollable. From there we look more broadly at a national effort as implemented in one state which leveraged clinical and regulatory experts to partner with nursing homes and disseminate emerging and evidence based practice cohorts to address the realities of the COVID-19 pandemic over an extended period. Then we move from the federal to two state level projects. One looks at the experience of embedding advanced practice nurses (APRNs) into long term care facilities in one state over a five year period including during COVID-19. The final presentation describes hands on support provided by one state government to nursing homes and assisted livings during COVID-19, including the coordination of staff testing and the implementation of the use of the antigen testing machines issued through federal policy. We conclude with a discussion of the interplay of federal, state, and local policy on nursing home experiences in COVID-19 and recommendations for more effective policy interventions.

